# Potential and Limitations of Large Language Models for Medical Literature Analysis: A Preliminary Investigation

**DOI:** 10.7759/cureus.92590

**Published:** 2025-09-17

**Authors:** Takahiro Kamihara, Takuya Omura, Atsuya Shimizu

**Affiliations:** 1 Department of Cardiology, National Center for Geriatrics and Gerontology, Obu, JPN; 2 Department of Endocrinology and Metabolism, National Center for Geriatrics and Gerontology, Obu, JPN

**Keywords:** co-occurrence network, large language model, medical literature analysis, pubmed database, text mining

## Abstract

Objective

While Large Language Models (LLMs) show great promise for various medical applications, their black-box nature and the difficulty of reproducing results have been noted as significant challenges. In contrast, conventional text mining is a well-established methodology, yet its mastery remains time-consuming. This study aimed to determine if an LLM could achieve literature analysis outcomes comparable to those from traditional text mining, thereby clarifying both its utility and inherent limitations.

Methods

We analyzed the abstracts of 5,112 medical papers retrieved from PubMed using the single keyword "text mining." We used Google Gemini 2.5 (Google Inc., Mountain View, CA, USA) and instructed it to extract distinctive words, concepts, trends, and co-occurrence network concepts. These results were then qualitatively compared with those obtained from conventional text mining tools, VOSviewer and KH Coder.

Results

Google Gemini appeared to conceptually aggregate individual words and identify research trends. The concepts for co-occurrence networks also showed visual similarity to the networks generated by the traditional tools. However, the LLM’s analytical output was based on its own unique interpretation and could not be directly compared with the statistically derived co-occurrence patterns. Furthermore, since this study relied on a visual comparison of network diagrams rather than rigorous quantitative analysis, the conclusions remain qualitative.

Conclusion

Google Gemini indicated an ability to extract keywords, concepts, and trends. A co-occurrence network visually similar to those generated by conventional text mining tools was created. While it showed particular strengths in conceptual summarization and trend detection, its limitations - including its black-box nature, reproducibility challenges, and subjective interpretations - became apparent. With a proper understanding of these constraints, LLMs may serve as a valuable complementary tool, with the potential to accelerate literature analysis in medical research.

## Introduction

The remarkable progress in Large Language Models (LLMs) in recent years suggests their diverse applicability within the medical domain [1-3]. These applications encompass, but are not limited to, analyzing patient complaints [4], assessing pre-surgical risks [5], and reviewing extensive medical literature [6]. However, alongside this rapid development, the inherent challenges of applying LLMs to rigorous academic analysis in medicine have been noted - particularly their black-box nature and the difficulty of reproducing results.

Conversely, text mining has long been an established methodology for evaluating and extracting information from written content [7-10]. However, mastering text mining is a time-consuming process. Against this background, our study attempted to validate the initially optimistic hypothesis of whether an LLM could be used by a novice to achieve an outcome comparable to that of an expert using traditional text mining. Through this validation, we aimed to clarify the utility of LLMs in literature analysis while also highlighting their inherent limitations.

This study analyzed medical papers on "text mining" using an LLM and compared its findings with those derived from conventional text mining tools. For this validation, we selected biomedical literature containing the term "text mining," indexed in PubMed.

## Materials and methods

This study involved the analysis of publicly available data and was therefore exempt from review by the Ethics and Conflict of Interest Committee of the National Center for Geriatrics and Gerontology. As this study did not involve the direct use of personal data, approval for the use of LLMs, including Google Gemini, was granted by the Director of the Department of Cardiology at the National Center for Geriatrics and Gerontology. In accordance with institutional regulations, an official application was submitted to the Chief Information Security Officer (submission date: December 28, 2023), and approval for continued use was granted through December 27, 2024.

This study was conducted in accordance with the PRISMA-ScR (Preferred Reporting Items for Systematic Reviews and Meta-Analyses: Scoping Review Extension) guidelines to ensure the transparency of our methods. A PubMed search was performed on July 30, 2025, using the single keyword “text mining,” yielding 5,112 documents. These documents were subsequently converted into text files, while preserving both the PubMed and Abstract formats for subsequent analysis. All data obtained from this search were included in the analysis, and the correctness of the data extraction was verified by three independent researchers. The extracted items included PMID, publication date, title, DOI, abstract, author names, affiliations, MeSH terms, and keywords. Since all documents were included, we have opted not to present a flowchart; instead, the total number of documents and the search date are explicitly stated within the Materials & Methods section. In accordance with the PRISMA-ScR guidelines, we have also included a discussion on the methodological limitations of our study.

For the LLM analysis, we utilized Google Gemini 2.5 (Google Inc., Mountain View, CA, USA). The stored text files were provided as input to the LLM, which was instructed to analyze the following aspects: (i) identification of distinctive words and conceptual clusters within the corpus, (ii) elucidation of prevailing research trends, and (iii) conceptual design of a co-occurrence network diagram to visually represent word co-occurrence relationships in the literature. The specific prompts provided to the LLM were: "Please analyze the attached text file for distinctive words, concepts, and trends," and "Please create a co-occurrence network from the following text file."

The figure was generated using Mermaid 11.9.0 (MIT-licensed software, Copyright (c) 2020-2023 Knut Sveidqvist, publicly available at https://github.com/knsv/mermaid). This software is provided free of charge and grants permission to anyone who obtains a copy to use, copy, modify, merge, publish, distribute, sublicense, and/or sell copies of the software without restriction. These rights also include permission to allow others to whom the software is provided to do the same, without limitation.

In parallel, text mining analyses were conducted using VOSviewer version 1.6.20 [11] and KH Coder version 3.Beta.07f [12]. With VOSviewer, keyword density visualization was performed by including all keywords from the 5,112 collected documents and generating a co-occurrence network diagram using the software’s Density visualization function. Additionally, VOSviewer was used to construct co-occurrence networks directly from the full abstracts of the documents. KH Coder was similarly employed to analyze the abstracts and generate corresponding co-occurrence networks.

Finally, the analytical results from Google Gemini were systematically compared with those obtained from VOSviewer and KH Coder to identify commonalities and discrepancies, as well as to explore the limitations and unique characteristics of the LLM.

## Results

**Figure 1:**
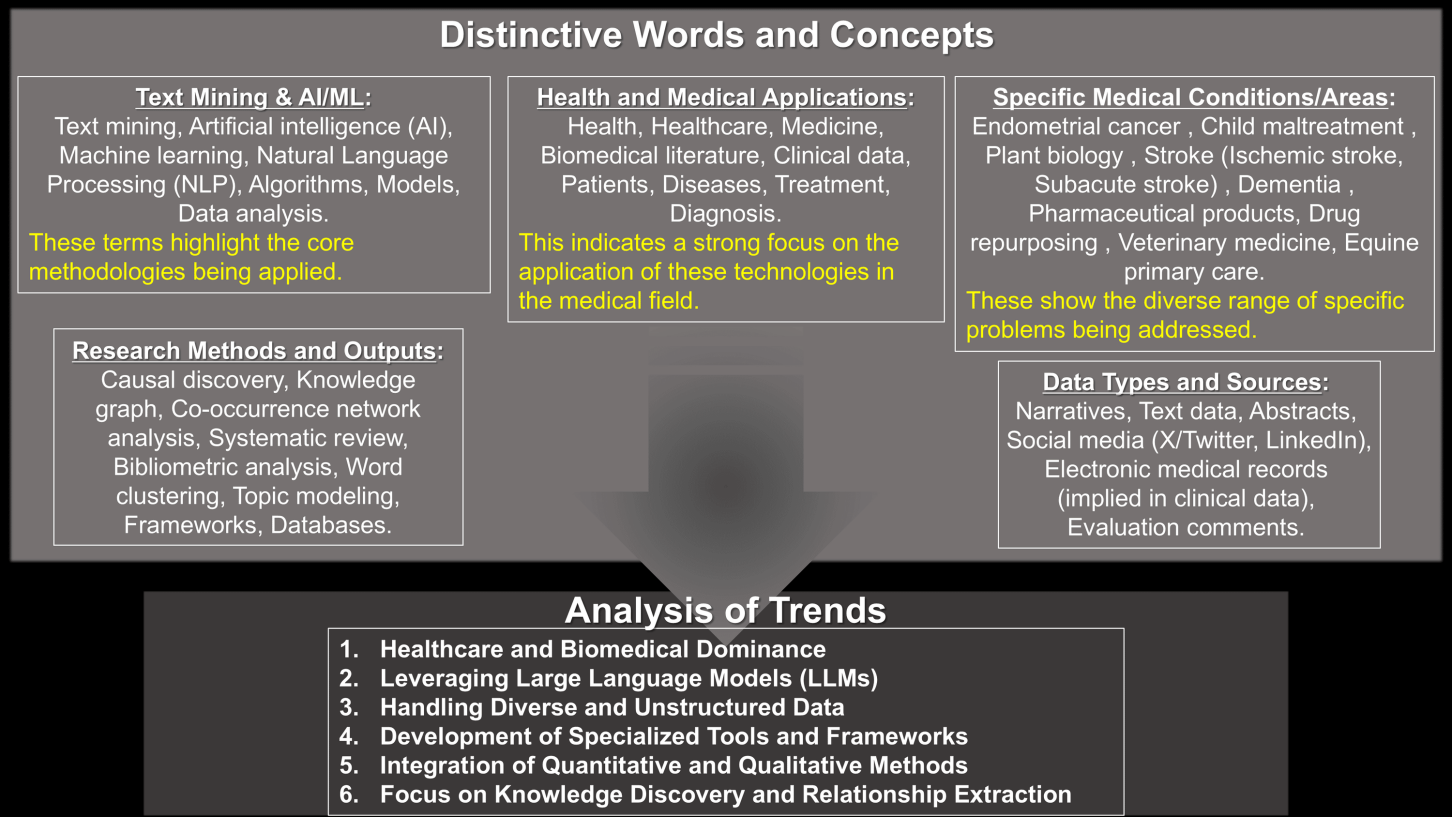
Conceptual and Trend Analysis of "Text Mining" Literature Using Large Language Model This figure summarizes the analytical outcomes derived from 5,112 documents retrieved via a PubMed search for "text mining," subsequently converted into PubMed Abstract format text files, and then processed by Google Gemini for Distinctive Words and Concepts and Trends. Google Gemini demonstrated the capability not only to extract individual words but also to aggregate them into coherent concepts and to discern prevailing trends subjectively.

Analysis of the abstracts of 5,112 "text mining"-related documents from PubMed using Google Gemini revealed that the LLM not only extracts individual words, but also effectively groups them into concepts (Distinctive Words and Concepts) and identifies research trends (Figure 1).

**Figure 2:**
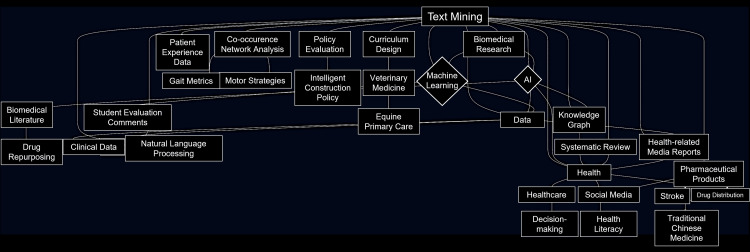
Co-occurrence Network Diagram Generated by Large Language Model This figure was generated using Mermaid 11.9.0 (https://github.com/knsv/mermaid) based on the co-occurrence network diagram concepts formulated by Google Gemini. These concepts were derived from 5,112 documents obtained through a PubMed search for "text mining," which were converted into PubMed Abstract format text files.

Furthermore, Google Gemini generated concepts for co-occurrence network diagrams, which were subsequently constructed using Mermaid 11.9.0 (Figure 2).

**Figure 3:**
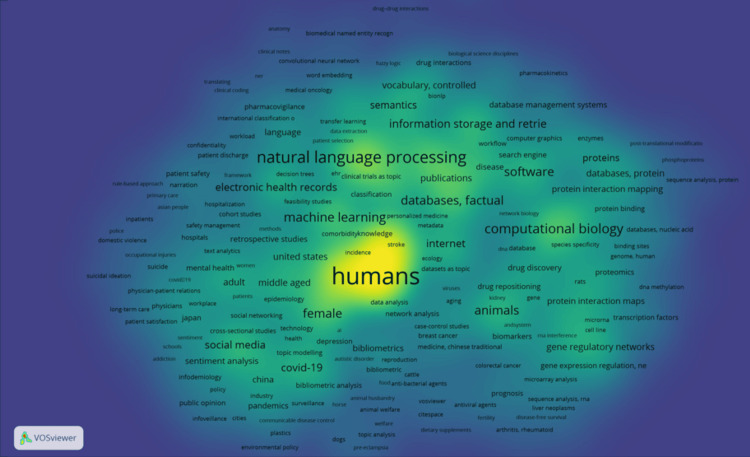
Keyword Density Visualization Using Text Mining This figure presents a density visualization created with VOSviewer [11], utilizing 5,112 documents obtained from a PubMed search for "text mining" and converted into PubMed format text files. All keywords were selected as targets for analysis. The visualization reveals a frequent focus on "human"-related research, with terms such as "machine learning," "computational biology," and "natural language processing" appearing in its vicinity, thereby enabling the identification of research trends.

Subsequently, VOSviewer was employed to visualize the keyword density of the 5,112 documents. This visualization indicated a frequent focus on "human"-related research, with terms such as "machine learning," "computational biology," and "natural language processing" appearing in close proximity, thereby facilitating the identification of prevailing research trends (Figure 3).

**Figure 4:**
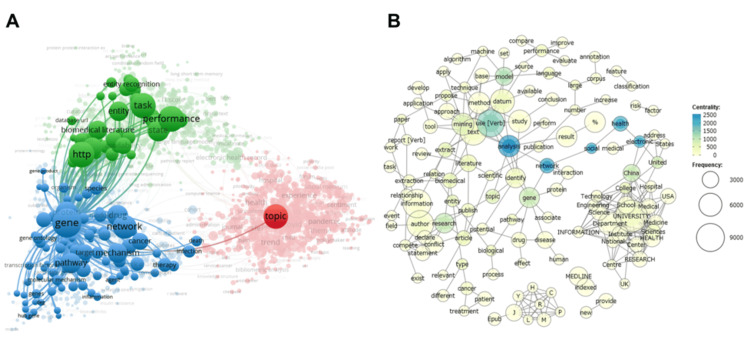
Comparison of Co-occurrence Networks Generated by Text Mining Tools A) This figure illustrates a co-occurrence network, constructed by VOSviewer [11] through text analysis of 5,112 documents retrieved from a PubMed search for "text mining" and converted into PubMed Abstract format text files. A prominent network is observed around the term "gene." B) This figure displays a co-occurrence network generated by KH Coder [12] from the analysis of 5,112 documents obtained from a PubMed search for "text mining" and converted into PubMed Abstract format text files. The term "gene" is central to this network. Additionally, "large" and "language" are positioned in the upper-right quadrant, suggesting a connection to Large Language Models. In this figure, words exhibiting higher centrality are represented by darker-colored circles.

Following this, VOSviewer was utilized for a comprehensive text analysis, extending beyond mere keywords to generate a co-occurrence network. This analysis demonstrated the formation of a substantial network primarily centered around the term "gene" (Figure 4A). Additionally, KH Coder was applied to analyze the text and construct a co-occurrence network. The word "gene" consistently appeared at the network's core. Moreover, the terms "large" and "language" were positioned in the upper right quadrant, suggesting an association with LLMs (Figure 4B).

The co-occurrence network diagram concepts presented by Google Gemini (Figure 2) showed a certain degree of correspondence with the diagrams generated by VOSviewer and KH Coder (Figures 3, 4A, 4B) when subjectively compared regarding major themes and associations. However, the specific labeling of these concepts by Google Gemini was based on its unique interpretative framework, and its results cannot be strictly compared with the statistically derived co-occurrence patterns provided by traditional text mining tools (Figures 3, 4).

## Discussion

This study subjectively compared the results of medical literature analysis conducted by Google Gemini against those obtained from conventional text mining tools VOSviewer and KH Coder, in order to validate its limitations. These tools have been extensively used in numerous studies [13-17].

Google Gemini indicated the capability to not only extract individual words, but also to conceptually aggregate them as "Distinctive Words and Concepts," based on its unique interpretation (Figure 1). This finding suggests the possibility that LLMs possess the ability to infer higher-order semantic relationships - a contrast to traditional text mining tools, which primarily extract patterns based on word co-occurrence and frequency (Figures 3, 4). Furthermore, the ability of an LLM to analyze "Trends" appears to be valuable for rapidly comprehending the overarching direction of research.

Regarding the conceptualization of co-occurrence network diagrams, a visual similarity was observed between the network diagram presented by Google Gemini (Figure 2) and those generated by VOSviewer and KH Coder (Figures 3, 4), concerning central themes and primary associations. However, this congruence was not based on a strict statistical evaluation. For instance, the central role of "gene" in the co-occurrence network diagrams from VOSviewer and KH Coder showed an interpretative alignment with Google Gemini's conceptualization of "biomedical literature" as a subgraph. Similarly, keywords highlighted by VOSviewer, such as "human," "machine learning," and "natural language processing," subjectively aligned with Google Gemini's concept of "text mining research methods and applications." This degree of alignment may indicate that the capabilities of LLMs are approaching those of conventional text mining tools, but the fundamental differences in their analytical methodologies must be considered. These findings are in agreement with prior studies conducted in other disciplines [18]. Furthermore, emerging literature in the medical domain suggests that Gemini may be more useful than other tools, supporting the appropriateness of our decision to utilize Gemini in this study [19].

LLMs offer the advantage of articulating analysis results in natural language and summarizing them as concepts, thereby enhancing information accessibility for non-specialists. However, a caveat exists: the labeling of concepts generated by LLMs is rooted in their unique understanding, which can occasionally render the precise interpretation of their meaning challenging when compared to the statistically derived co-occurrence patterns provided by traditional text mining tools (Figure 1). Moreover, given the substantial disparity in information volume between Figure 2 and Figures 3, 4, the ability of LLMs to appropriately modulate information granularity remains a pertinent future challenge.

As a secondary finding, the predominant themes within the "text mining"-related medical papers analyzed in this study, as illustrated by VOSviewer’s density visualization (Figure 3), centered on "human"-related research - particularly its integration with technologies such as "machine learning," "computational biology," and "natural language processing." This reflects the current landscape, where text mining is an indispensable tool for analyzing complex human-derived biomedical data, including clinical, genomic, and epidemiological datasets. Furthermore, the central appearance of "gene" in the co-occurrence network diagrams from VOSviewer and KH Coder (Figures 4A, 4B) clearly demonstrates the broad application of text mining in gene-related research, such as analyzing gene expression data, identifying gene-disease associations, and genome annotation. This underscores text mining’s critical role in bioinformatics. Text mining has indeed been effectively utilized in numerous bioinformatics studies [20-23]. Additionally, the proximity of terms like "large" and "language" to "gene" in the KH Coder diagram suggests that LLMs themselves are gaining traction as research tools used in conjunction with text mining within contemporary medical research, thereby reinforcing the relevance of this study’s chosen theme.

Limitations

This study is significant in demonstrating the potential of LLMs to extract critical information from medical text data at a level comparable to - or even more conceptually nuanced than - conventional text mining tools. The natural language processing capabilities of LLMs could potentially accelerate the process by which researchers efficiently locate necessary information within vast literature and uncover novel insights. However, this study is subject to several significant limitations.

Firstly, the LLM employed was exclusively Google Gemini, precluding a performance comparison with other LLM models. Given that LLM performance varies across models [24,25], further comparative studies are requisite for drawing generalized conclusions. Secondly, it remains an open question whether the LLM's presented concept of "biomedical literature" can be strictly linked to bioinformatics, which was represented by the "gene"-centric word clusters extracted via text mining. Thirdly, the "accuracy" of the LLM's analysis results was subjectively compared. This study was not based on rigorous quantitative analysis but on a visual comparison of network diagrams, and thus, the conclusions are purely qualitative. Therefore, the presentation of quantitative results remains a challenge for future research.

Finally, the most critical limitation of this study is that the protocol for generating figures with LLMs is entirely a black box. We used Google Gemini 2.5, a commercial LLM, which represents the most critical constraint of our research. Commercial LLMs are black-box systems with non-disclosed internal workings - including training data, model parameters, and frequent updates - which makes scientific reproducibility difficult at present. Specifically, even with the publication of our prompts and analytical procedures, other researchers would find it challenging to replicate the exact same results. Additionally, our search strategy may have included some irrelevant literature. It is crucial to note that this study demonstrates the utility of LLMs as a methodological tool for grasping academic trends, not as a replacement for a rigorous, systematic literature review. At this stage, it is not possible to directly use an LLM to conduct a systematic review.

## Conclusions

This study suggested that Google Gemini is capable of extracting key terms, fundamental concepts, and meaningful trends from medical literature. A co-occurrence network, visually similar to those generated by conventional text mining tools, was created. Notably, the LLM exhibited particular strength in conceptual summarization and trend detection, which suggests its potential for higher-order inferential reasoning beyond the statistical associations typically revealed by traditional methods.

The LLM's performance in deriving analytical insights from biomedical literature was qualitatively comparable to that of established text mining approaches. However, a key characteristic of LLMs is their tendency to assign labels to terms and concepts based on internal interpretive mechanisms. This context-dependent labeling can sometimes obscure the intended semantic meaning. When this limitation is properly understood and managed, LLMs may significantly accelerate the process of literature analysis in medical research. These findings highlight the potential for LLMs to evolve into valuable tools for a broad range of healthcare professionals, offering enhanced efficiency and depth in textual data interpretation.
